# Do Registered Dietitians, Nutrition Students, and Laypeople Perceive Individuals with Obesity Differently?

**DOI:** 10.3390/ijerph18178925

**Published:** 2021-08-25

**Authors:** Giovana Santarosa Cassiano, Joana Pereira Carvalho-Ferreira, Nicola J. Buckland, Diogo Thimoteo da Cunha

**Affiliations:** 1 Multidisciplinary Food and Health Laboratory, School of Applied Sciences, State University of Campinas, Limeira 13484-350, Brazil; gi_scassiano@hotmail.com (G.S.C.); joana.ferreira@fca.unicamp.br (J.P.C.-F.); 2 Department of Psychology, University of Sheffield, Sheffield S10 2TN, UK; n.buckland@sheffield.ac.uk

**Keywords:** weight bias, weight stigma, antifat attitudes, counseling, antiobesity, stigma, causes of obesity

## Abstract

(1) Background: Obesity is associated with significant social consequences, and individuals with obesity are regularly affected by weight-related stigmatization experiences. This study compares antifat attitudes among registered dietitians (RD), nutrition students, and laypeople and assesses which factors related to the perceived causes of obesity influence these attitudes. (2) Methods: An online survey was conducted in Brazil with RD (*n* = 336), nutrition students (*n* = 300), and laypeople (*n* = 403) with questionnaires assessing antifat attitudes and perceived causes of obesity. (3) Results: All groups presented low antifat attitudes. Minor differences in antifat attitudes were found among the three groups. Compared to RDs and nutrition students, laypeople presented higher Weight Control/Blame scores, but with a small effect size (η^2^ = 0.01). Weight bias was predicted by age, sex, and body mass index. External, social, and financial factors were not perceived to be very important in the development of obesity by RD and students. (4) Conclusions: Since slight differences were seen among RD and students compared to laypeople, and some perceptions of the causes of obesity indicate a stigmatized view. It is essential to place a greater focus on educating and updating these health professionals and students about weight stigma and its consequences for the mental and physical health of individuals.

## 1. Introduction

Over the past decades, overweight and obesity rates have increased worldwide [1,2]. In 2019, 30.2% of women and 22.8% of men in Brazil were categorized in the obesity range according to Body Mass Index (BMI) [3]. Obesity is associated with significant social consequences, and individuals with overweight and obesity are regularly affected by weight-related stigmatization experiences [4], commonly known as weight stigma. Weight stigma is the social rejection and devaluation of those who do not comply with prevailing social norms of adequate bodyweight and shape [5]. This “socially” adequate body is regarded as slim and fit [6]. The weight stigma and bias can manifest in many ways, through negative beliefs, attitudes, stereotypes, preconceived judgments, verbal assaults, physical stigma, and physical barriers and obstacles, due to someone’s weight [7]. Several decades of research have established the presence and widespread nature of societal weight-based stereotypes. These stereotypes include assumptions that people with overweight are lazy, lacking in willpower and self-discipline, incompetent, unmotivated to improve their health, non-compliant with medical treatment, and are personally to blame for their higher bodyweight [4]. The social acceptability of these stereotypes often leads to overt forms of prejudice and even discrimination. People with overweight or obesity can experience weight-based discrimination in nearly every area of life, including relationships, inequities in employment and hiring process, disadvantages in education, and disparities in healthcare [4].

Surprisingly, healthcare is a setting where weight stigma is particularly pervasive, with significant consequences for patients’ mental and physical health [8]. Weight bias has been observed among nurses, medical doctors, medical students, trainee dietitians, and dietitians [9,10]. The stigma is often justified to motivate behavior change and promote healthier behaviors [11]. However, research has consistently documented the link between weight stigma and health consequences, including maladaptive eating, disordered eating behaviors, the risk for eating disorders, decreased motivation to engage in physical activity, vulnerability to depression and anxiety, physiological stress, and weight gain [12,13,14]. Therefore, weight stigma has been considered a psychosocial contributor to obesity, due to these links between experiences of weight stigma and weight-related behaviors and health [14]. There are two main theoretical frameworks that attempt to explain the link between weight stigma and health. The cyclic obesity/weight-based stigma model [15] suggests that weight stigma contributes to weight gain in a cycle: Stigma is characterized as a stressor that provokes psychological (shame, stress appraisals), behavioral (increased eating), and physiological (e.g., elevated cortisol) responses, which may contribute to weight gain, triggering the cycle again. Other authors propose a social identity threat model, suggesting that weight stigma threatens the social identity of people with overweight and obesity, increasing stress and motivation to escape situations where stigma is anticipated (e.g., physical activity and healthy eating), while reducing self-regulation [11]. Together, these psychological and physiological mechanisms may negatively affect health and promote weight gain [11].

Health professionals, including dietitians, can be a common source of stigma for patients with overweight [16]. Individuals report feeling ignored and mistreated in clinical settings [17]. Patients with overweight also believe that their clinicians would prefer not to treat them [18]. As a result, patients with a higher BMI report avoiding healthcare because of the discomfort of being stigmatized [19,20]. Dietitians and nutrition students also hold negative attitudes and prejudice toward people with obesity (PWO) [10,21], which is problematic, since many of them work, or will work, directly with PWO at the various levels of health care. In Brazil, the proportion of PWO aged 20 years or older doubled, while the proportion of people with overweight rose from 43.3% to 61.7% in the last 17 years [3]. These are alarming numbers, but as evidenced, experiencing weight stigma can be damaging to health. Although it has been shown that dietitians and nutrition students can have negative attitudes towards PWO [21,22], the magnitude of these attitudes compared to laypeople in Brazil is unknown. Understanding this magnitude and the contexts that PWO are exposed to the deleterious effects of weight stigma (i.e., general social environment and clinical settings), can inform targeted interventions at the individual and collective levels.

It is expected that dietitians and nutrition students hold fewer negative attitudes than laypeople, since they have greater knowledge about the multifactorial etiology of obesity [23]. However, similar results between groups may indicate that the stigma of obesity is widespread and not related to scientific knowledge. Moreover, weight bias can vary between different societies; for instance, a multinational study compared weight bias among adults of the United States (US), Iceland, and Canada, and undergraduate students of Hawaii, Australia, Iceland, and the United States [24]. In the adult samples, medians of Universal Measure of Bias-FAT version (UMB-FAT).scores differed significantly, indicating larger heterogeneity and density for scores in the US sample compared with Canada and Iceland. Meanwhile, in the student sample, the lowest scores were observed among Icelandic students, and the highest scores among students from the US. Thus, weight stigma can differ between countries, due to their sociodemographic differences and population perceptions. Therefore, this study compares antifat attitudes among Brazilian registered dietitians (RD), nutrition students, and laypeople, and assesses which factors related to the perceived causes of obesity influence these attitudes.

## 2. Literature Review

Negative attitudes and weight stigma towards PWO perpetuates health inequity, and impairs interventions designed to address this critical public health issue [25]. Placing blame for weight gain solely on the individual conflicts with the World Obesity Federation’s classification of obesity as a disease that requires collaborative action by health professionals, policy-makers, and industry [25,26]. Substantial evidence has demonstrated that healthcare professionals, including physicians, nurses, medical students, physical therapists, exercise professionals, and nutrition professionals, hold stereotypes and negative attitudes toward patients with obesity [10,27,28,29].

Regarding RD, evidence also shows negative attitudes towards PWO, including studies carried out in Brazil [21,22,30,31]. In a systematic review [28], three of four cross-sectional studies examining weight bias among dietitians documented the occurrence of weight bias. For instance, Edelstein et al. [31] found that 76% of RD (*n* = 128) had a strong-to-moderate preference for thin people in comparison with fat people, which far exceeded the results from the general population (52%). Similarly, Cori, Petty, and Alvarenga [21] found a high prevalence of weight bias reported by dietitians in Brazil. They attributed negative characteristics to PWO, such as greed (67.4%), unattractiveness (52%), ungainliness (55.1%), lack of willpower (43.6%), and laziness (42.3%). Another study demonstrated that dietitians (*n* = 49) had an unfavorable evaluation of a person with overweight compared to a person with normal weight [32]. Meanwhile, in another study, dietitians exhibited an ambivalent attitude toward clients with an overweight BMI. Frequency data indicated that dietitians attributed overweight to emotional problems, and that their clients were unable to set realistic goals for weight loss, but they were ambivalent about clients’ ability to follow a weight reduction diet or an exercise program [33]. These antagonistic results demonstrate that RD’s attitudes still need to be further explored.

When assessing weight bias in nutrition or dietetic students, studies showed a moderate level of fatphobia, and higher self-reported BMI predicted lower fatphobia among the participants [10,34]. In a controlled research, 182 undergraduate dietetic students were randomly assigned to read one of four mock health profiles of patients who varied in weight-related characteristics and sex. They were asked to make judgments about the patient’s health status and treatment adherence. The dietetic students rated patients with obesity as being less likely to comply with treatment recommendations and having poorer diet quality and health status than patients without obesity [30]. However, the relationship between weight bias and nutrition students is unclear. In two studies, there were no differences in attitudes towards PWO in dietetic students versus non-dietetic; and nutrition versus non-nutrition students [32,35]. Interestingly, in the first study, the results showed positive attitudes towards PWO in both groups [32]. Oberrieder et al. [36] compared implicit weight bias of dietetic undergraduate students and RD. Both groups showed negative obesity attitudes, with no differences between the two groups. However, students who perceived themselves as having a healthy weight had a higher negative attitude than participants who self-reported being overweight [36].

Finally, weight stigma in a sample of laypeople was evaluated in a multinational examination of weight bias, including populations of four countries (United States, Canada, Iceland, and Australia). According to BMI, over half of the adult population was classified as overweight or obese in each country [24]. Despite the high prevalence rates of obesity, weight bias was present in these countries, and it was associated with blame attributions and personal responsibility for bodyweight. For instance, attributing obesity to behavioral causes, lack of willpower, and personal responsibility predicted stronger weight bias. To date, no study has compared antifat attitudes and weight bias between laypeople and professionals in Brazil.

It is imperative to identify the motivations and drivers for these negative attitudes. Just identifying the phenomenon is insufficient for proactive action to minimize the weight stigma. The association between an obesogenic and stigmatizing environment and professionals with negative attitudes towards PWO can aggravate the overweight situation in the country. The magnitude of weight stigma is especially relevant when considering Brazilians food choice motives and difficulties in identifying unhealthy food items [37,38]. Furthermore, as weight stigma can vary between different cultures [24], studies in different countries can help define nation-specific recommendations, and local policies focusing on specific cultural factors.

## 3. Materials and Methods

### 3.1. Sample, Design, and Data Collection

Data were collected using the online platform Google Forms (Alphabeth Inc. Mountain View, CA, USA) from July to December 2020. Only Brazilians older than 18 years old were enrolled. A non-probability purposive, with chain-referral sampling, was employed. Due to the coronavirus pandemic, all participants were recruited through social networks (Facebook, Instagram, WhatsApp, and e-mail). The sample had three different groups: Laypeople, RD, and nutrition students. The minimum sample was established considering three groups, effect size f = 0.15, alpha error of 5%, and power of 90%. Calculations showed 188 participants were required in each group.

Since the study population is large, accessible and online research was employed, an increased sample number of *n* > 300 was desired for laypeople and *n* > 250 for each group of RD and nutrition students to reduce sampling error and increase heterogeneity. To identify non-attentive participants, some questions had a reverse coded answer. Moreover, the standard deviation (SD) between the Antifat Attitudes Test (AFAT) variables were checked for each participant. Twelve participants were excluded for presenting SD = 0. All participants signed an informed consent electronically.

### 3.2. Measures

The AFAT, with 34 items, was applied in the three groups to measure negative attitudes towards individuals with obesity. The instrument was developed by Lewis (1997) [39] and adapted to Brazilian Portuguese by Obara and Alvarenga (2018) [40], who described the cross-cultural adaptation and validation. The three subscales are: (1) “Social and character depreciation” (15 items), ascribing socially undesirable personality characteristics and social disregard for PWO; (2) “physical/romantic unattractiveness” (10 items), reflecting perceptions that PWO are clumsy, unattractive and are unacceptable as romantic partners; and (3) “weight control/blame” (9 items) that assesses beliefs concerning whether PWO is responsible for their weight. Responses were given on a five-point Likert-type scale (1 = strongly disagree to 5 = strongly agree). Higher scores reflect more negative attitudes towards individuals with obesity, and scores above 3.0 were considered antifat bias. A total composite score and the subscales’ scores were calculated using the mean value of all items.

For further investigation, RD and nutrition students answered two more questionnaires after the AFAT, one about the causes of obesity and the other about characteristics attributed to PWO. These two questionnaires are part of an instrument that was translated, adapted, and tested in Brazil with RD [21], based on international works [27,41], which enrolled dietitians and primary care physicians on their research, respectively. Laypeople did not answer these questionnaires because they were only administered to health professionals. To assess beliefs about the causes of obesity, RD and nutrition students rated the relevance of 17 factors as part of obesity etiology. These items included biological (e.g., genetic factors) and behavioral (e.g., overeating) causes. Items were rated using a five-point Likert-type scale (1 = not at all important to 5 = extremely important).

The attitudes about the personal characteristics of individuals with obesity, in turn, were evaluated using twelve semantic differential items. A five-point Likert-type scale was anchored at each end by two opposing personal characteristics, such as “lazy” vs. “hardworking”. Following the model of Foster et al. (2003) [27], for each item, respondents used the five-point scale to indicate where they placed individuals with obesity along the continuum.

Sociodemographic data, such as age, sex, educational level, and income, were assessed at the end of the survey as control variables. RD and nutrition students were also asked when they started higher education to measure both the average period that students were in college and the years of work as RD. Self-reported weight and height data were collected to verify the BMI (weight/height^2^). Four individuals with severe obesity (BMI > 45kg/m^2^) were excluded to reduce bias.

### 3.3. Analysis

All variables were analyzed for theoretical distribution by analyzing the means, SD, a distribution histogram, using a Kolmogorov–Smirnov test (with the Lilliefors correction) to test the compliance to a normal distribution. The AFAT scores were normalized using log transformation. The results were presented in their original non-transformed values for clarity.

The one-way ANOVA with Welch correction was used to compare means among the three groups. The Games-Howell post hoc test was used for multiple comparisons. The t-Student’s test was used to compare two independent groups. The homoscedasticity was checked using Levene’s test. Partial eta squared (η^2^) was used to verify the effect size of the one-way ANOVA tests. This effect size was classified as small (η^2^ = 0.01), medium (η^2^ = 0.06), or large (η^2^ = 0.14) [42]. Cohen’s d was used to verify the effect size of t-statistics. The effect size (d) was classified as small (d = 0.2), medium (d = 0.5), and large (d = 0.8) [42].

Principal component analysis (PCA) with Promax rotation was used to extract significant factors of the causes of obesity questionnaire. It was considered only items with factor loading >0.50. Kaiser–Meyer–Olkin and Bartlett’s sphericity tests were used to check the adjustment of PCA. The composite reliability (CR) higher than 0.70 was used to ascertain the factor’s reliability.

A multiple linear regression model was used to determine which variables were associated with AFAT scores (normalized). The independent variables in the model were those variables that presented Pearson’s correlation r > 0.20. The variables were included using forward selection. The model goodness-of-fit was checked through the residual analysis. For all variables, *p* < 0.05 was considered significant. Data are presented as means (SD) unless specified. Statistical analyses were performed using Statistical Package for Social Sciences (SPSS) v.20 (IBM Corp. Armonk, NY, USA) and SmartPLS v3.2.8 (SmartPLS GmbH, Bönningstedt, Germany). There were no problems with missing data.

## 4. Results

### 4.1. Comparing Laypeople with RD and Nutrition Students

The total sample composed of 1,039 individuals, divided into laypeople (*n* = 403), registered dietitians (*n* = 336), and nutrition students (*n* = 300), and their sociodemographic characterization can be seen in Table 1. Laypeople were mainly female (64%), aged 32.05 (11.93) years, and with a mean BMI of 26.29 (6.34) kg/m^2^. Most were engaged in (33.7%) or had completed (25.3%) higher education, and 40.9% (*n* = 165) had a family income higher than five Brazilian minimum age (up to US$951.00).

RDs were primarily female, with an average (SD) age of 32.54 (9.12) years old and BMI 24.72 (4.21) kg/m^2^. Most (56.8%) were postgraduate, and 13.4% had a master’s degree. Most of them (46.7%) had a family income higher than five Brazilian minimum age (>US$951.00). Nutrition students were also primarily female, with a mean age of 25.12 (7.20) years old and BMI of 24.04 (4.33) kg/m^2^. Most nutrition students had a family income between two and five Brazilian minimum wages (between US$380.40 and US$951.00). Laypeople had a significantly higher BMI compared to RD and students (*p* < 0.05). Students were significantly younger than RD and laypeople (*p* < 0.05).

Table 2 shows the results of the AFAT scales. Only the “Weight Control/Blame” subscale had significant differences between groups, with laypeople showing higher averages than RD and Nutrition students. However, the size effect was small (η^2^ = 0.01). Men presented a higher composite score (*p* = 0.01) and weight control/blame (*p* < 0.001) than females.

Table 3 shows the comparative models (linear regression) among laypeople, RD, and nutrition students of AFAT constructs and composite scores. It is possible to observe different independent variables with different effects across the models. In general, with increasing age, there was an increase in antifat attitudes, especially for laypeople. A higher income also affected antifat attitudes negatively, i.e., as income increased, antifat attitudes decreased, but only for laypeople. Females also had fewer antifat attitudes than men. This effect was observed for all groups in model 4. Finally, in models 3 and 4, an increased BMI was significantly linked with lower antifat attitudes for RD, but BMI was not significantly linked with antifat attitudes in students or laypeople. Education affected laypeoples’ antifat attitudes, i.e., as the education level decreased, antifat attitudes increased.

### 4.2. Investigating RD and Students’ Beliefs about the Causes of Obesity

The results of the causes of obesity questionnaire can be seen in Table 4. Scores of the items “low self-esteem”, “repeated dieting”, and “personality” were higher in RD perception compared to students. However, all effect sizes were small (d = 0.22; d = 0.21, and d = 0.16, respectively).

After the factor analysis, five factors were extracted: factor 1 (items: 3, 4, 5); factor 2 (items: 7, 14); factor 3 (items: 1, 2, 6, 12); factor 4 (items: 9, 11, 17); factor 5 (items: 8, 10, 13, 15, 16). The factors were named based on item characteristics (Table 4). Factor 1 (higher energy consumption) was the factor with the highest average, i.e., higher energy consumption seems to be the most important factor for developing obesity, according to RD and nutrition students. After, biological factors and psychological and behavioral factors (with no difference between them) were the second most perceived relevant factors for developing obesity. Factor 4 (external factors) and 5 (complacency), respectively, were the factors that contribute less to developing obesity according to RD and nutrition students. Nutrition students attributed more importance to biological factors (factor 2) than RD, but the difference had a small effect size (d = 0.26).

Table 5 presents linear models for RD and students using the perceived causes of obesity as independent variables and the AFAT composite score as the dependent variable. For both groups, the more participants rated complacency (factor 5) as important for developing obesity, the greater the antifat attitudes according to the AFAT. However, some differences were found between the models. For RDs, antifat attitudes were negatively affected by biological, external, and psychological and behavioral factors, whereas, for students, this effect was only observed for biological factors.

The results on the characteristics attributed to PWO are shown in Table 6. In all characteristics, responses tended to be towards the positive qualities. Only the first two items (Sweet Tooth × Controlled and Not attractive × Attractive) had averages close to 2.5, but this does not indicate negative attitudes. Furthermore, the first two items were the only ones with significant differences between groups, with RDs’ scores being higher (more positive) than those of nutrition students. However, the effect size of this difference was small (d = 0.16). The factor analysis extracted two factors: factor 1 (1, 2, 3, 4, 5, 8, 11) and factor 2 (7, 9, 10, 11, 12). Comparing these two factors, factor 2 showed a higher average than factor 1 in both groups.

## 5. Discussion

### 5.1. General Discussion

The main objective of this study was to investigate whether there were differences between laypeople, RD, and nutrition students concerning antifat attitudes, and the possible drivers for the antifat attitudes. In general, laypeople showed a slightly higher weight bias than RD and nutrition students, with small effect sizes. Moreover, being female, increasing BMI and age, lower education level, and placing greater importance on the controllable causes of obesity were linked to higher scores of antifat attitudes in at least one of the three groups. This is the first study comparing antifat attitudes among RD, nutrition students, and laypeople. However, more robust differences among groups were expected regarding weight bias. This result may be a consequence of the widespread characteristic of weight bias, present in various domains of life and coming from different groups, whether health professionals or not [4,43]. Several decades of studies have established the presence and pervasive nature of weight-based social stereotypes [4]. Authors say that weight bias remains socially acceptable, with PWO considered one of the last acceptable targets of denigration [16]. These negative perceptions persist, in part, due to societal beliefs that body weight is a matter of personal responsibility and willpower, even with considerable scientific evidence that genetic, emotional, and psychosocial factors are notable contributors to the development and maintenance of overweight and obesity [14,44]. Furthermore, this discrimination is rarely challenged, and sanctions to prohibit prejudice or discrimination based on weight do not exist [24].

In the current study, several sociodemographic factors affected antifat attitudes. Men demonstrated subtly higher antifat attitudes, which has also been observed in other studies. For instance, in a large sample of 66,799 volunteers from the US, men had higher implicit and explicit antifat bias than women [45]. In another study with medical students, gender was associated with students’ preferences for “thin” or “fat” people, with males being twice as likely as females to report a significant antifat bias [9]. Similarly, medical doctors, on average, showed strong implicit and explicit antifat bias, with females showing less implicit bias [46]. Women are more vulnerable to weight-related discrimination than men [47]. Consequently, they might be more sensitive to potential prejudices related to weight [24], which might be related to this sex difference found here and earlier.

We also found that a higher education level was associated with lower antifat attitudes among laypeople. This finding has previously been evidenced in a recent study with populations from different countries, in which greater weight bias was associated with having a lower education level [48]. It is worth mentioning that the relationship between weight bias and socioeconomic status might depend on divergent sociocultural perspectives. In some cultures, in which individual responsibility is described as the leading cause of self-fulfillment, health, and wealth, obesity can be seen as a self-inflicted condition. In other cultures, in which individuals’ situations are considered a result of various circumstances, obesity might not be seen as self-inflicted [49]. In these cultures, especially people with a high level of education may be aware of social barriers as determinants for self-fulfillment, wealth, and health, including bodyweight (35). This knowledge is consistent with the lower weight bias found in the present sample. Additionally, increases in age were also associated with higher antifat attitudes in all groups, especially in laypeople. This finding differs from an international study, in which greater weight bias was associated with being younger [50]. In the Brazilian population, obesity has increased in recent years, and society is more conservative than in the past, especially among the elderly. This is the first result that relates age and antifat attitudes in Brazil, so further investigation is needed in the future. Still, this initial finding suggests that antifat attitudes are highest in older adults.

Furthermore, it is unclear if BMI can be considered an antecedent of weight bias, since the results may be contradictory in the literature [43]. The present results showed diverse findings according to the different groups investigated. In the RD, weight bias was negatively predicted by BMI, such that a lower BMI was associated with greater antifat bias, and this finding is similar to previous research. Schwartz et al. [51] found that higher BMI was associated with lower scores on the Explicit Bias Scale and the Implicit Associations Test. Another study with health trainees revealed that higher self-reported BMI predicted lower fatphobia [10]. RD with overweight may have a better understanding of the reality of being a PWO. Moreover, the social identity theory suggests that people favor their social group over other groups. Therefore, people are less likely to hold prejudices against the group they identify with [52]. On the other hand, a high BMI did not significantly affect the composite score of laypeople or nutrition students. This weight bias expressed by them can partly result from an internalized stigma, making them believe that social stereotypes based on weight are personally applicable [24,53]. Alternatively, some people may view their higher bodyweight as a temporary condition that they can escape, thereby lessening the extent to which they perceive themselves as belonging to a “population with obesity.” Therefore, they see others with higher bodyweight as an out-group that may deserve blame [24,53].

This study also revealed interesting findings that may affect the adherence of PWO in RD counseling care. It was found that the strongly perceived causes of obesity in RD and nutrition students’ groups were energy consumption, biological and psychological aspects. Extrinsic and mainly financial and social factors were ranked as less important factors of obesity etiology. However, it is known that the interaction between genetic inheritances [54] and social and cultural factors [55] are relevant for the increasing prevalence of obesity. Therefore, the health professional focus should exceed the cited factors and include social and economic issues, which play a role in how one deals with food preferences, choices, and intake. One of the responsibilities of the Food and Nutrition Education (FNE) discipline—present in the Nutrition curriculum in Brazil—is to contribute to the deconstruction of the biological approach to nutrition, promoting a broader view of eating behavior, emphasizing the need to value the social, economic, and cultural aspects that involve food [23]. However, professors’ understanding of FNE, their work hours, and employment by private or public universities may be limited, affecting nutrition students’ education [23]. A limited notion of FNE may result in a decontextualized or inadequate education of future professionals impacting the development of abilities needed for professional activities, and probably, on the way they perceive obesity [56]. As a result, RD and nutrition students may not feel fully equipped to deal with behavioral and social issues that influence obesity [21].

Based on the data presented here of weight stigma in the health care setting (i.e., nutrition professionals and students) and considering its health consequences, it is essential to focus on interventions to improve the practice of health professionals in addressing individuals’ weight management behaviors [57]. When planning interventions, an existing review highlights the importance of having a holistic view, which includes the health professionals’ knowledge, skills, attitudes, values, and professional identity within the environment in which they work [57]. Moreover, future interventions to address obesity, including those designed to support health professionals with the provision of nutrition and physical activity counseling, should comprehensively address the issue of weight stigma [25]. This involves supporting health professionals to identify and understand their attitudes towards PWO and their beliefs regarding the etiology of obesity. As well as promoting a shift away from weight and appearance-focused treatment, and towards treatments that focus on optimal health and wellbeing, and establishes comfortable and non-stigmatizing environments where PWO feel welcome [57].

### 5.2. Practical Implications

In the current study, obesity stigma was similar between the groups. This result shows that obesity stigma can be widespread in the population, regardless of education and type of profession. Furthermore, the variables associated with AFAT were similar, indicating a homogeneous phenomenon. Obesity campaigns must be precise, reinforcing habits and practices (e.g., healthy eating, active life, professional counseling, etc.) in an empathetic and supportive way. Public policies must facilitate people’s access to a healthy and non-stigmatizing environment.

Together, these results also suggest the need to increase education and awareness of the weight stigma in Nutrition courses and professional updates. It is imperative to change the hostile social environment that PWO face and ensure that negative assumptions about them do not adversely influence follow-up practices. It is necessary to focus on interventions that challenge weight-based stereotypes and raise awareness of the consequences of weight stigma on mental and physical health, while encouraging active listening skills with empathetic communication. To attend this challenge, a multidisciplinary group of international experts developed a joint consensus statement with recommendations aiming to eliminate weight bias that can be useful for students, professionals, professors, and researchers [58]. It has been seen that people are influenced by others’ perceptions about PWO, especially if those people are of valuable reference groups [59]. Thus, a possible target for reducing stigma may be to provide students and professionals with information about the consequences of weight stigma and the importance of weight tolerance. That information could come from reference groups, professional associations, or admired colleagues to motivate them to identify with these groups that condemn antifat attitudes.

It is important to raise the awareness of students and professionals about the uncontrollable causes of obesity. Educators and professors should emphasize—in the FNE discipline—the importance of biopsychosocial and cultural aspects of eating practices, in order not to place all the blame for weight gain/maintenance on the individual. Finally, it is key to provide professionals and students with skills to deal with the behavioral causes of obesity, with a more in-depth study of eating behavior, and focusing on a more humanistic, respectful, and empathetic approach.

### 5.3. Limitations

This study has limitations. First, the sample was not selected randomly, as the survey was applied online, participants were selected for convenience, and those who chose to answer the questionnaire had access to the internet, were computer literate, and probably had a specific interest in the subject. Moreover, most of the three study groups were young, highly educated, with high family income, and female. This female predominance, especially in the groups of RDs (94.6%) and nutrition students (92.7%), was expected since nutrition is a profession dominated by women, as shown in a study by the Brazilian Federal Council of Nutritionists, in which 94.1% of the sample was female [60]. Similarly, other Brazilian studies with dietitians and nutrition students, had similar female predominance, with 97.1% and 93.7% of women, respectively [21,22]. This may have affected the results of this study as women seem to hold less weight-based discrimination than men [47], probably because they are more vulnerable to this type of discrimination and might be more sensitive to potential weight-related prejudices [24]. Despite being a potential bias considering socioeconomic aspects, the sample is compatible with Brazil’s population of nutrition students and professionals.

In this study, we used measures of explicit antifat attitudes, which are attitudes that people consciously recognize, obtained through self-report measures. However, self-reported attitudes may not predict behavior, and may be vulnerable to response bias and concerns about social desirability. Therefore, it would be interesting for future research in Brazil to use an instrument that measures the implicit antifat attitudes, such as the Implicit Association Test [61], since implicit measures would probably deepen the knowledge about antifat attitudes on these groups.

## 6. Conclusions

RD and nutrition students showed no major differences in antifat attitudes when compared to laypeople. The RD and nutrition students showed a stigmatized view of obesity in some perceived causes of obesity, which may interfere with the care, follow-up, and health of individuals with overweight and obesity. It is important that obesity-related campaigns should be focused on improving health and wellbeing, rather than focusing solely on weight loss, which can increase stigmatization. Meanwhile, it is imperative to place a greater focus on educating and updating health care professionals and students about weight stigma, its consequences for the stigmatized individual, and the role of uncontrolled causes in obesity. Such initiatives will tackle the stigmatizing and damaging environment that PWO encounter in health care settings.

## Figures and Tables

**Table 1:** Sample characteristics.

Variable	Laypeople *N* (%)	RD *N* (%)	Nutrition Students *N* (%)
*Gender*			
Male	145 (36)	18 (5.4)	22 (7.3)
Female	258 (64)	318 (94.6)	278 (92.7)
*Age*			
18–30 years	223 (55.3)	168 (50)	246 (82)
31–40 years	79 (19.6)	107 (31.8)	35 (11.7)
41–50 years	63 (15.6)	42 (12.5)	16 (5.3)
51–60 years	28 (6.9)	14 (4.2)	1 (0.3)
>61 years	7 (1.7)	3 (0.9)	0 (0.0)
Missing	3 (0.7)	2 (0.6)	2 (0.7)
*Level of Education*			
Read and write, complete primary education, incomplete primary education, and incomplete high school	13 (3.2%)	NA	NA
Complete high school	83 (20.6)	NA	NA
Incomplete higher education	136 (33.8)	NA	300 (100)
Complete higher education	102 (25.3)	92 (27.4)	NA
Postgraduate, Master’s, and Doctorate	69 (17.1)	244 (72.6)	NA
*Income*			
Less than half the minimum wage * and between half and one minimum wage	23 (5.7)	12 (3.6)	19 (6.4)
Between one and two minimum wage	50 (12.4)	34 (10.1)	49 (16.3)
Between two and five minimum wage	144 (35.7)	122 (36.3)	113 (37.7)
More than five minimum wages	165 (40.9)	157 (46.7)	96 (32.0)
Do not know	20 (5.0)	11 (3.3)	22 (7.3)
Missing	1 (0.3)	NA	1 (0.3)

* Brazil minimum wage in 2020: R$ 1045.00 (US$ 190.20); NA = not applicable. RD = Registered Dietitians.

**Table 2:** Mean; Standard Deviation (SD) of the antifat attitudes for laypeople, registered dietitians (RD), and nutrition students as measured with the Antifat Attitudes Test (AFAT).

AFAT Subscales	Laypeople (Mean; SD)	RD (Mean; SD)	Nutrition Students (Mean; SD)
Subscale 1-Social/Character disparagement (CR = 0.78)	1.27 a; 0.3	1.25 a; 0.3	1.22 a; 0.3
Subscale 2-Physical/Romantic unattractiveness (CR = 0.84)	1.73 a; 0.5	1.80 a; 0.5	1.72 a; 0.5
Subscale 3-Weight Control/Blame (CR = 0.84)	2.08 a; 0.7	1.95 b; 0.6	1.96 b; 0.6
Composite score (CR = 0.72)	1.62 a; 0.4	1.60 a; 0.4	1.56 a; 0.4

a, b—homogenous groups according to *Games-Howell* multiple tests (*p* < 0.05), to be read horizontally. Possible scores ranged from 1 = strongly disagree to 5 = strongly agree. CR = Composite reliability. AFAT = Antifat Attitudes Test.

**Table 3:** Comparative models (linear regression) among laypeople, registered dietitians, and nutrition students of the Antifat Attitudes Test constructs and composite scores.

Models, and Dependent and Independent Variables	Laypeople		RD		Nutrition Students	
	Beta	*p*	Beta	*p*	Beta	*p*
*Model 1: Social/character disparagement*						
Age	**0.22**	**<0.001**	**0.15**	**0.005**	**0.15**	**0.007**
Income (1 = higher income)	**−0.12**	**0.01**	0.007	0.89	0.006	0.92
*Model 2: Physical/romantic unattractiveness*						
Age	**0.28**	**<0.001**	**0.24**	**<0.001**	**0.15**	**0.01**
Sex (1 = female)	−0.10	0.71	**−0.15**	**<0.001**	**−0.20**	**0.001**
BMI	**−0.017**	**0.04**	**−0.21**	**0.003**	**−0.15**	**0.01**
*Model 3: Weight control/blame*						
Age	**0.28**	**<0.001**	**0.17**	**0.002**	-	ns
Sex (1 = female)	**−0.14**	**0.005**	**−0.14**	**0.009**	-	ns
BMI	0.02	0.67	**−0.18**	**0.001**	-	ns
Income	**−0.14**	**0.003**	0.02	0.63	-	ns
*Model 4: Composite score*						
Age	**0.32**	**<0.001**	**0.22**	**<0.001**	**0.17**	**0.005**
Sex (1 = female)	**−0.10**	**0.02**	**−0.15**	**0.004**	**−0.14**	**0.01**
BMI	0.02	0.57	**−0.18**	**0.001**	−0.11	0.06
Education level (1 = high education level)	**−0.18**	**<0.001**	−0.02	0.36	0.00	1.00

Bold values = significant effects *p* < 0.05; ns = non-significant. RD = Registered Dietitians. BMI = Body Mass Index.

**Table 4:** Causes of obesity according to registered dietitians (RD) and nutrition students in Mean, Standard Deviation (SD).

	RD		Nutrition Students	
Causes	Mean; SD	4 + 5 (%)	Mean; SD	4 + 5 (%)
1—Physical inactivity	4.5; 0.8	87.8	4.6; 0.8	85.6
2—Emotional and mood changes (depression, anxiety)	4.7; 0.6	93.1	4.7; 0.6	96.3
3—Food addiction	4.7; 0.7	92.0	4.8; 0.5	95.7
4—Overeating	4.5; 0.8	89.0	4.5; 0.8	87.6
5—Eating inappropriate food	4.3; 0.9	80.6	4.4; 0.9	81.3
6—Low self-esteem	4.3; 0.9	80.1	4.2; 1.0	77.7
7—Metabolic-hormonal changes	4.5; 0.7	88.4	4.7; 0.6	93.6
8—Lack of willpower	3.5; 1.3	53.0	3.6; 1.3	58.0
9—Extrinsic factors (family, friends, environment, media)	4.2; 0.9	74.4	4.3; 0.9	79.3
10—Do not consider being overweight a problem	3.9; 1.2	66.7	3.9; 1.2	66.0
11—Increased availability of food and portions sold and consumed	4.1; 1.0	74.5	4.1; 1.0	71.7
12—Repeated dieting	4.1; 1.0	73.5	4.1; 1.0	70.3
13—Lack of awareness about your weight	3.8; 1.1	61.6	3.8; 1.2	63.0
14—Genetic factors	3.9; 1.0	61.9	4.1; 0.9	70.7
15—Like to eat a lot	3.6; 1.2	53.3	3.4; 1.2	47.3
16—Personality	3.1; 1.2	35.4	3.0; 1.3	32.6
17—Financial or social situation	3.4; 1.2	44.6	3.5; 1.2	48.0
Factor 1—High energy consumption (CR = 0.800)	4.5 ^A^; 0.6	-	4.5 ^A^; 0.6	-
Factor 2—Biological factors (CR = 0.838)	4.2 *^,B^; 0.7	-	4.4 *^,B^; 0.6	-
Factor 3—Psychological and behavioral factors (CR = 0.779)	4.3 ^B^; 0.6	-	4.3 ^B^; 0.7	-
Factor 4—External factors (CR = 0.773)	3.9 ^C^; 0.7	-	3.9 ^C^; 0.7	-
Factor 5—Complacency (CR = 0.857)	3.6 ^D^; 0.9	-	3.6 ^D^; 0.9	-

* *t*-student test for groups with *p* < 0.05 (RD and students); Repeated measures ANOVA for factors; ^A^, ^B^, ^C^, ^D^—heterogenous groups according to *Bonferroni* multiple tests (*p* < 0.05), to be read vertically for each factor. Possible scores ranged from 1 = not at all important to 5 = extremely important.

**Table 5:** Comparative models (linear regression) among registered dietitians (RD) and nutrition students of the Antifat Attitudes Test (AFAT) composite scores.

Model Dependent and Independent Variables	Beta	*p*
*Model 1: RD—AFAT composite score*		
Age	**0.12**	**0.02**
BMI	−0.09	0.07
Sex (1 = female)	**−0.12**	**0.01**
Factor 2—Biological factors	**−0.19**	**0.001**
Factor 3—Psychological factors	**−0.12**	**0.03**
Factor 4—External factors	**−0.13**	**0.02**
Factor 5—Complacency	**0.48**	**<0.001**
*Model 2: Nutrition students—AFAT composite score*		
Age	**0.11**	**0.03**
BMI	−0.09	0.10
Sex (1 = female)	**−0.12**	**0.02**
Factor 2—Biological factors	**−0.25**	**<0.001**
Factor 5—Complacency	**0.41**	**<0.001**

BMI = Body Mass Index. RD = Registered Dietitians; Bold values = significant differences *p* < 0.05; BMI was used as an adjusting variable. AFAT = Antifat Attitudes Test.

**Table 6:** Registered dietitians (RD) and nutrition students’ attitudes about people with obesity in Mean, Standard Deviation (SD), and Frequency of responses.

		RD		Nutrition Students	
Paired	Attributes	Mean and SD	4 + 5 (%)	Mean and SD	4 + 5 (%)
1—Sweet Tooth	Controlled	2.7; 0.7	3.9	2.5; 0.9	7.3
2—Not attractive	Attractive	2.9; 0.8	17.0	2.8; 1.0	16.3
3—Clumsy	Elegant	3.2; 0.8	24.7	3.1; 0.9	23.3
4—Not determined	Determined	3.2; 0.8	25.6	3.1; 0.9	23.7
5—Lazy	Hardworking	3.1; 0.8	17.6	3.0; 0.9	12.0
6—Untidy	Tidy	3.2; 0.8	24.4	3.2; 1.0	25.4
7—Rebel	Compliant	3.7; 0.8	25.0	3.8; 0.9	59.4
8—Uninteresting	Interesting	3.4; 0.9	33.3	3.4; 0.9	36.6
9—Sad	Happy	3.1; 0.9	24.7	3.1; 1.0	26.3
10—Dishonest	Honest	3.8; 0.9	48.4	3.9; 0.9	55.6
11—Not compromised	Involved	3.2; 0.9	28.0	3.3; 1.00	32.3
12—Unpleasant	Pleasant	3.8; 0.8	50.6	3.8; 0.9	54.6
Factor 1—Laziness related (CR = 0.894)		3.1 ^B^; 0.6	-	3.0 ^B^; 0.7	-
Factor 2—Emotional related (CR = 0.860)		3.5 ^A^; 0.6	-	3.6 ^A^; 0.7	-

^A^, ^B^—heterogenous groups according to *paired t*-tests (*p* < 0.05), to be read vertically for each factor. Scores were not collected for laypeople.

## Data Availability

The data presented in this study are available on request from the corresponding author. The data are not publicly available, due to being part of a broader study in progress.
